# Prospects of Endovenous Laser Ablation (EVLA) Standardization—Mid-Term Results of a Four-Zone Dosimetry Guiding Tool for 1940 nm Laser

**DOI:** 10.3390/jcm12134313

**Published:** 2023-06-27

**Authors:** Abhay Setia, Slobodan Dikic, Sahit Demhasaj, Thomas Schmitz-Rixen, Ronald Sroka, Claus-Georg Schmedt

**Affiliations:** 1 Department of Vascular Surgery, Diakonie-Klinikum, 74523 Schwaebisch Hall, Germanyclaus-georg.schmedt@diakoneo.de (C.-G.S.); 2 Division of Vascular and Endovascular Surgery, Department of Vascular Medicine, Klinikum Darmstadt, 64283 Darmstadt, Germany; 3 Department of Vascular Surgery, German Society of Surgery, Langenbeck-Virchow-House, Luisenstr. 59, 10117 Berlin, Germany; 4 Laser-Forschungslabor, LIFE Center, University Hospital, Ludwig-Maximilian University, 81377 Munich, Germany; 5 Department of Urology, University Hospital, Ludwig-Maximilian University, 80539 Munich, Germany

**Keywords:** EVLA, 1940 nm, flush placement, radial fibre, four-zone guiding tool

## Abstract

Background: Heterogeneity regarding dosimetry and reporting of endovenous laser ablation (EVLA) mandates the development of a standardized protocol. This study presents the mid-term results of EVLA with 1940 nm-laser and radial-fibre, supported by a four-zone dosimetry tool. Materials and methods: Four anatomical dosimetry zones for great saphenous veins (GSV) and two for small saphenous veins (SSV) were defined with set power levels. Zone-1G (4 W) extended from the inguinal ligament to the apex of femoral triangle, Zone-2G (4 W) from the apex of femoral triangle to the upper border of patella. Zone-3G (3 W) from the patella to the tibial tuberosity. Zone-4G (2 W) extended from the tibial tuberosity to the ankle. Zone-1S from the sapheno-popliteal junction to the tibial tuberosity. Zone-2S from the tibial tuberosity to the ankle. Power was increased by 1 W for veins >10 mm and decreased by 1 W when fibre sticking was encountered. Pullback-velocity was max. 1 mm/s. Results: A total of 152 consecutive patients (185 procedures) were recruited. Mean follow-up time was 11.9 months. Mean linear endovenous energy density for GSV was Zone-1G:42 J/cm, Zone-2G:33 J/cm, Zone-3G:27 J/cm, Zone-4G:22 J/cm, Zone-1S:34 J/cm, Zone-2S:27 J/cm. Occlusion rates were 98.9% (1-month) and 93.7% (12-months). Complications at 1 M were low, namely laser-induced paraesthesia (LIP) 2.2% and endovenous heat-induced thrombosis (EHIT) 1.6%. Persistent LIP (12 M) was observed in 0.5%. Conclusion: The proposed four-zone guiding tool is a step towards standardizing dosimetry and documentation for EVLA with 1940 nm. This strategy shows good mid-term results with minimal complications. Long-term follow-up and application in further centres are necessary to prove its reproducibility. Such a guiding tool could improve the ability to analyse, compare and review different EVLA wavelengths and fibre types.

## 1. Introduction

Endovenous laser ablation (EVLA) in recent years has observed a trend towards using longer wavelength laser systems in combination with a radial-emitting fibre [1,2,3,4,5,6,7,8,9,10,11,12,13,14]. Because of a higher coefficient of absorption in water for 1940 nm as compared to other wavelengths [15,16,17,18], and radial dissipation of the light energy with radial emitting fibres, the desired ablation of the vein wall can be achieved with lower power levels with thermal changes predominantly limited to the intima and media [19,20]. This translates in a clinical setting into high occlusion rates, low postoperative pain and low complication rates [14,21].

Even though ablation with 1470 nm and 1940 nm laser systems are established methods of EVLA, discrepancies still exist regarding the power levels and dosimetry adjustments for both 1940 nm [8,9,10,11,12,13,14], and 1470 nm laser [5,6,7]. The current literature reveals two dosimetry strategies, the first being constant power levels irrespective of vein diameter for both 1940 nm and 1470 nm laser [5,6,10,12]. The second strategy considers the variable vein diameter throughout its length and recommends varying power levels depending on the vein diameter for, e.g., increasing power levels by 1 watt for every 1 mm increase in vein diameter [7,14]. Although both these strategies are currently being practiced, these do not account for other anatomical factors, such as proximity to skin or nerves. These findings persuade the development of a tactical dosimetry approach considering the anatomical dynamics. Additionally, a recent literature review revealed considerable heterogeneity regarding the documentation and reporting of EVLA (for, e.g., stump length, status of accessory veins), making a result comparison in the frame of a meta-analysis unfeasible [21].

This study presents a conceptualized four-zone dosimetry guiding tool and its application for EVLA with 1940 nm laser system and radial light application with its short and mid-term results.

## 2. Materials and Methods

In this prospective, non-randomized, observational study, consecutive patients were recruited between 1 June 2017 and 30 July 2019. The EVLA procedures performed in the operation theatre complex of the Department of Vascular Surgery, DIAK Klinikum, Schwaebisch Hall, Germany. Written consent was obtained from the patients for the procedure and for pseudonymous data collection. The study was approved by the ethics committee of the hospital and University Heidelberg (S-082/2016). The study included all patients aged >18 years with primary varicose veins with incompetent truncal veins (GSV/SSV) with an outer diameter (OD) of ≤13 mm. OD > 13 mm was accepted only in patients with small, localized dilatations that involved up to 3 cm segments of the truncal vein. All diameters mentioned in this study correspond to the outer diameters (OD), until unless specified. Patients suffering from, or with a history of, thrombophlebitis of the GSV or SSV, deep vein thrombosis, previous varicose vein surgery and those not willing to take part in the study were excluded.

Patients’ characteristics like demographics, the CEAP classification, revised Venous Clinical Severity Score [22], vein characteristics and intra- and postoperative details were documented by vascular surgeons on a custom-made standardized protocol and were tabulated (Microsoft Excel, Redmond, USA). VCSS were filled by the examining vascular surgeon during the clinical visits. Strict adherence to the guidelines of the European general data protection regulations (EU-DSGVO2016/679; Europäische Datenschutz-Grundverordnung) was ensured.

EVLA Equipment: The EVLA procedure was carried out with a thulium fibre laser system (VelaXL, Starmedtec GmbH, Starnberg, Germany) emitting at 1940 nm, in combination with a radial emitting fibre system (Saturn Side Fibre, 600 µm/400 µm Radial-Fibre, Light-Guide-Optics, Meckenheim, Germany) and the detailed steps of the operative procedure are described elsewhere [14]. Intraoperative monitoring was performed with duplex ultrasound (LogiQ-e, GE Healthcare, USA). Standardized pre- and postoperative duplex ultrasound was performed with LogiQ-S8, GE Healthcare, USA.

The duplex ultrasounds were performed in a standing position. Reflux was defined as a retrograde flow for more than 500 ms on provocation at the sapheno-femoral or sapheno-popliteal junction. Additionally, outer diameter (OD) measurements were performed at 7 predefined points for GSV (G1–G7) and 5 for SSV (S1–S5) [14]. The protocol was improved a few months after the beginning of the study, to document the status of the accessory veins (AVs) with respect to their identifiability, presence/absence of reflux and OD measurements (3 cm peripheral to the SFJ) [23].

**Figure 1:**
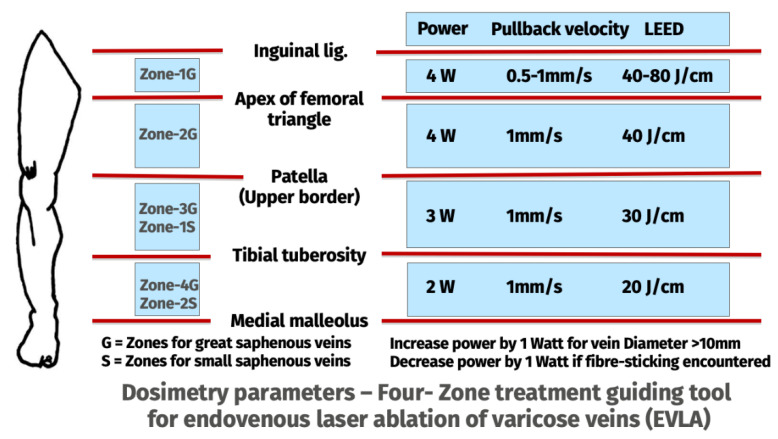
Four-Zone treatment guiding tool to support surgeons’ dosimetry decision with 1940 nm laser system and radial fibre. LEED: Linear endovenous energy density.

**Figure 2:**
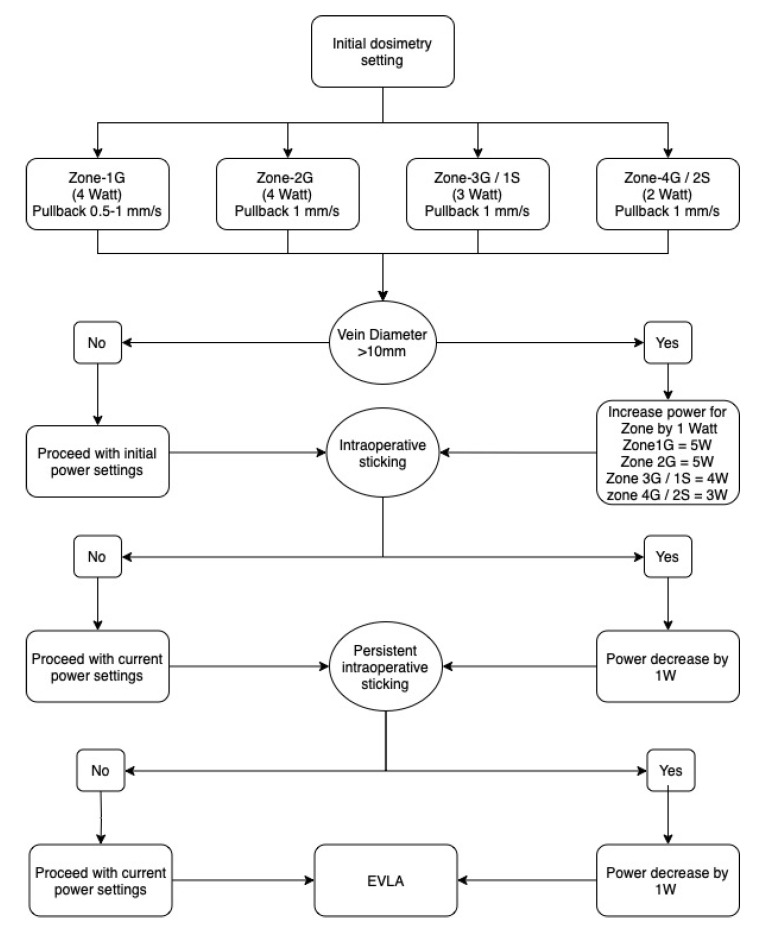
Flowchart to direct the dosimetry decision according to the four-zone treatment guiding tool. Decrease in power levels due to sticking was allowed twice. Zone-1G,2G,3G,4G are zones for great saphenous veins and Zone-1S,2S for the small saphenous veins.

EVLA Dosimetry was guided by a custom-made four-zone guiding tool, depicted in Figure 1. Taking anatomical landmarks into account, four anatomical topographical zones for the lower extremity were considered. Zone-1G extended from the inguinal ligament to the apex of the femoral triangle (corresponding to the level of the perineum), Zone-2G from the apex of femoral triangle to the upper border of patella. Zone-3G from the patella to the tibial tuberosity. Zone-4G extended from the tibial tuberosity to the ankle joint. Based on this, the GSV was divided into four zones (Zone-1G–4G). Since the SPJ lies in the popliteal fossa, the SSV was divided into two zones. Zone-1S extended from the SPJ to tibial tuberosity and Zone-2S extended from the tibial tuberosity to ankle joint. Tactile feedback and the phenomenon of the fibre sticking were considered a trigger for decreasing the power levels by 1 W at any point of ablation. Power reduction of >2 W was not allowed (Figure 2). EVLA procedural details were documented for each zone individually. This standardized protocol is available as Supplementary Materials to this document.

**Figure 3:**
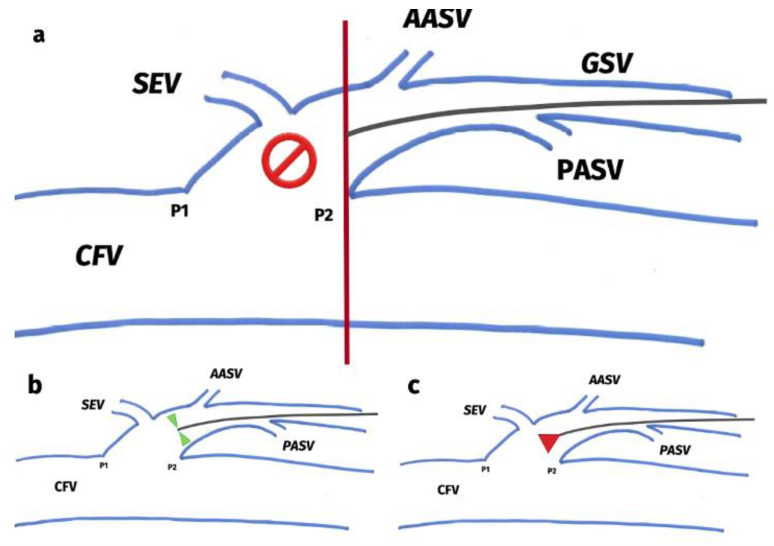
Schematic representation of ultrasound guided placement of the radial emitting fibre tip at the confluence to the deep vein. (**a**): The limit of proximity of the radial fibre to the deep vein is marked by an imaginary vertical line (depicted red here) passing through the distal ostial point (P2). To avoid damage to the deep veins, radial fibre placement beyond this line (no parking zone sign) is not recommended. (**b**): Radial energy dissipation from the radial fibre (denoted by green triangles) allows safe placement at the junction of deep vein, which, when performed with bare fibre (forward energy dissipation, denoted as red triangle), (**c**) might cause damage to the deep vein. SEV: superficial epigastric vein; AASV: anterior accessory saphenous veins; PASV: posterior accessory saphenous veins; CFV: common femoral vein; GSV: great saphenous vein; P1: proximal ostial point at the junction to the deep vein; P2: distal ostial point at the junction to the deep vein.

Radial-fibre tip placement was ‘‘flush,’’ and was defined as the ultrasound-guided, precise placement of the radial fibre tip at the distal ostial point (P2) at SFJ/SPJ in Trendelenburg position, as depicted in Figure 3. This was confirmed after completing tumescence injection and repositioned if necessary. The fibre selection and core diameter (600 µm/400 µm) were recorded and dependent on the OD, and were at the surgeons’ discretion.

**Figure 4:**
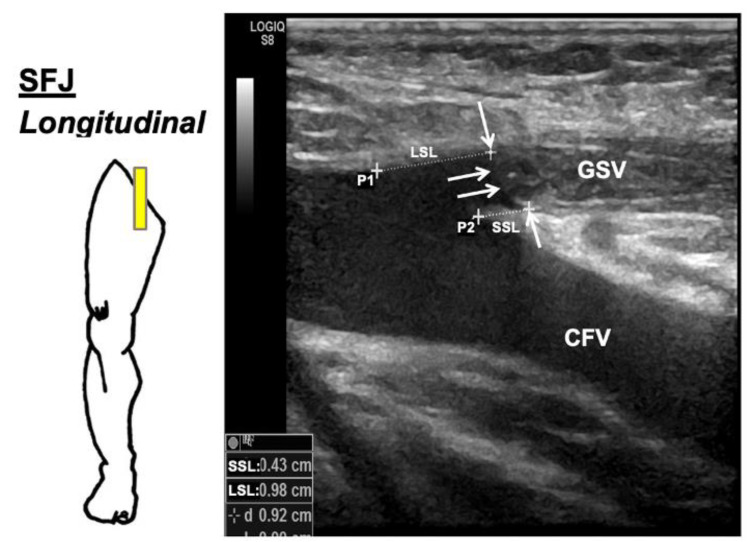
Postoperative ultrasound findings at the sapheno-femoral junction (SFJ) after EVLA with 1940 nm and radial fibre. The figure to the left is a schematic representation of the technique of ultrasound examination with the patient in standing position and the ultrasound probe held longitudinally (denoted by yellow rectangle). The arrows mark the central limit of the thrombus in the occluded veins. There is no involvement of the SFJ, and no damage to the deep veins is evident. Longest stump length (LSL): maximum distance between the SFJ/SPJ and the most peripheral part of the non-occluded vein stump. Shortest stump length (SSL): shortest distance between the SFJ/SPJ and the occluded vein. GSV: occluded great saphenous vein; CFV: common femoral vein; P1: proximal ostial point at the junction to the deep vein; P2: distal ostial point at the junction to the deep vein.

Follow-up clinical and duplex examinations were performed within 1 month (1 M) and at/after 12 months (12 M). Apart from postoperative complications [14], vein diameter and vein occlusion or reflux, the postoperative duplex ultrasound included the documentation of the following supplementary parameters. Longest stump length (LSL): maximum distance between the SFJ/SPJ (P1, Figure 4) and the most peripheral part of the non-occluded vein-stump. Shortest stump length (SSL): the shortest distance between the SFJ/SPJ (P2; Figure 4) and the occluded vein. Non-occlusion (NO) was defined as a longest stump length (LSL) >3 cm with compressibility of treated vein segment in the immediate postoperative period (up to 4 weeks). This was defined as early anatomical failure. The presence of reflux (Rx) after provocation in these segments was documented as well (NORx). Recanalization (RC) was defined as the reopening of an initially occluded/thrombosed vein at any time in the follow-up period, and was defined as late EVLA anatomical failure. This was checked for reflux as well and, if present, was documented as RCRx. Patients with either NO or RC were categorized as EVLA anatomical failure. Endovenous heat-induced thrombus (EHIT) was classified and treated accordingly [24] (Supplementary Figure S1).

Statistical analysis: Mean, range and median were calculated using computer software (Microsoft Excel V16.41). An ANOVA test was used for repeated measures, unpaired T-test and Fischer exact test were used to determine the statistical significance, and a *p*-value of *p* < 0.05 was considered to be significant. Percentage change in OD was calculated at 1 month and 12 months as compared to the preoperative OD.

## 3. Results

Patients’ characteristics and CEAP classification are listed in Supplementary Table S1. The treated collective comprised of 152 patients (female to male ratio 1.53) with 185 truncal veins (GSV 147, SSV 38). Of these, both limbs were operated on in 25/152 patients, and in 8/152 patients the GSV and the SSV were treated simultaneously. All the patients were examined postoperatively, and the average follow-up time was 11.9 months (1–25 months) after the index procedure. The follow-up rate in terms of procedures/veins treated was 100% (185/185) at 1 month and 85.9% (159/185) at 12 months. The median preoperative VCSS was 6 (2–22).

**Figure 5:**
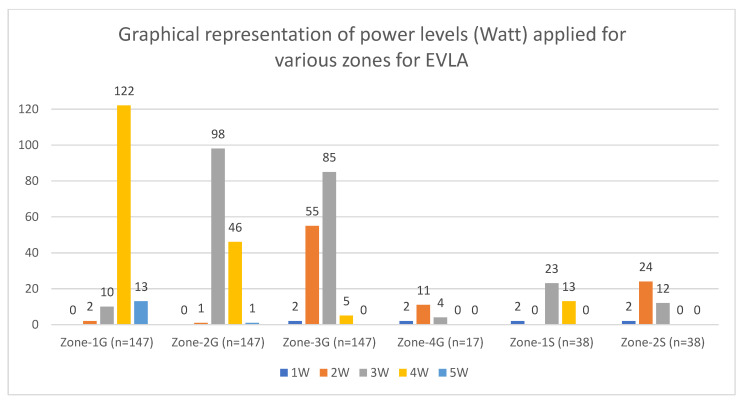
Graphical representation of power levels (watt) applied for various zones for EVLA. Power reduction was instigated by the sticking phenomena, which was predominantly observed in Zones-2G (*n* = 99/147) and -3G (*n* = 57/147).

The intraoperative and dosimetry parameters are summarized in Table 1. Ultrasound-guided flush application of radial fibre tip was achieved in all the patients, and no intraoperative complications were observed. Zone-1G,2G and 3G and Zone-1S,2S were treated in all the GSVs and SSVs, respectively. Zone-4G was treated in 11.6% (17/147) of GSVs (Figure 5). In Zone-2G, sticking was observed in 67.3% (99/147) of GSVs, and in these the power was reduced (Figure 5), leading to lower median power (3 W) as compared to the initial setting (4 W).

An attempt to identify the accessory veins was performed in 117 limbs; AASV was identified in 88/117 (75%) limbs, and PASV in 66/117 (56%) limbs (Supplementary Table S2). AASV was incompetent in 22/88 (33.3%) limbs, and PASV in 3/66 (4.5%) limbs. All the incompetent AVs were treated. Intraoperative trials to puncture the AV to obtain endovenous access and navigation of the fibre through the vein were feasible in 16/22 (72.7%) AASV and in 1/3 PASV, and these were subjected to EVLA. Failed vein puncture and tortuous course of the vein hindering fibre advancement were the reasons to treat the AVs with conventional phlebectomy. Mini-phlebectomies for varicose branch veins were performed in all the patients with documentation of the number and location of incisions in each procedure (Supplementary Table S3). Crossectomy without stripping of the GSV was performed only in two cases with incompetent AASV (2/22) and a separate confluence with the deep vein. Ligature of perforating veins accompanied 23/185 (12.4%) EVLA procedures.

*Short-term (1 month) and mid-term (12 months) results* are summarized in Table 2 and Table 3. A reduction in the median rVCSS was observed at 1 month (rVCSS = 2) and at 12 months (rVCSS = 2). Regarding postoperative complications within 1 month (Table 3), laser-induced paraesthesia (LIP) [14] was observed in 4/147 (2.7%) GSV EVLA limbs in the innervation areas of the saphenous nerve below knee. This regressed in 3 patients at 12 months, and was persistent below knee in 1/147 (0.7%) patient. Within 1 month mechanically induced paraesthesia (MIP) [14] was present in four patients in regions of mini-phlebectomy incisions accompanying EVLA procedures of the GSV. This was persistent in 1/147 (0.7%) patient. No LIP 0/38 (0%) or MIP 0/38 (0%) were observed with the treated SSV. Within 1 month, mechanically induced hematomas (10/185; 5.4%) were observed in the regions of mini-phlebectomy incisions and involved the thigh (5/185; 2.7%) and lower leg (5/185; 2.7%). All of these had regressed at the 12-month follow-up. EHIT was observed in 3/185 (1.6%) EVLA procedures within 1 month. All these patients received therapeutic anticoagulation and were followed-up weekly (Supplementary Table S4).

All early anatomical EVLA failures (non-occlusions; NO = 2/185;1.1%) were seen in great saphenous vein. At 12 months, 8/159 (5%) of the followed-up EVLA procedures were developing recanalization (RC), 6/126 (4.7%) occurred in GSV and 2/33 (6%) in SSV. Persistent occlusion was seen in 149/159 (93.7%) of the truncal veins. All patients with early or late anatomical failure were asymptomatic, and required no invasive treatment.

The two groups, EVLA anatomical failure (either NO or RC) and EVLA anatomical success, were compared for factors responsible for failure. Multivariate analysis revealed no significant association between recanalization and pre-operative vein diameters in Zone-1 (*p* = 0.34), LEED Zone-1 (*p* = 0.24), type of fibre (600 vs. 400 micrometre *p* = 0.39) or age (*p* = 0.88). On the other hand, a higher BMI (>30.5 kg/m [2]) was associated with increased rate of anatomical failure (NO or RC) (*p* = 0.026). Mean BMI in patients with anatomical failure and anatomical success was 31 ± 3.5 kg/m [2] vs. 26.8 ± 5.4 kg/m [2], respectively. Since, the number of EVLA anatomical failures was low, these results are to be interpreted with caution.

## 4. Discussion

Unlike radiofrequency ablation (RFA), the availability of broad array of protocols, generators and fibres for EVLA contributes to the lack of a standardized modus operandi [21]. A recent meta-analysis revealed this problem, and reported pooled occlusion rates of 92% after EVLA, irrespective of wavelengths, fibres and energy levels applied [25]. Further, the current LEED recommendations are non-specific with heterogenous dosimetry strategies, and do not regard anatomic factors like diameter changes, proximity to the nerves or skin or sticking of the fibre [2]. With this paper, we approach the problem of lack of standardization in EVLA from two aspects. The first aspect being the standardized documentation of pre-, intra- and postoperative parameters that would enable the comparison of different wavelengths and different dosimetry protocols. Our work presents these parameters that are important for planning, execution and follow up of EVLA patients. It lays the foundations for a consensus based standardized operating procedure and documentation.

The second aspect is the four-zone model; the segmental execution of EVLA with respect to anatomical characteristics for a particular vein segment. Since the OD of the truncal veins decreases as one proceeds caudally [14,21] (Table 2), more power would be required to cause effective ablation in the proximal zones as compared to distal zones (Zone-1G, Zone-2G vs Zone-3G, Zone 4G for GSV and Zone-1S vs Zone-2S for the SSV). Another rationale for using higher power levels in proximal zones is to effectively occlude the junction of the merging GSV tributaries and emphasizes to avoid neo-reflux and reflux in the accessory veins. Moreover, the peripheral GSV/SSV lie in close vicinity of their respective nerves, and higher power may cause damage to the perivenous nerves. Hence, lower power was assigned in distal zones (Zones-3G and -4G for GSV, and Zone-2S for SSV). Increasing the power levels in larger vein diameters, >10 mm, was based on experience [7,14], that larger veins would require more energy to achieve adequate thermal damage. Power reduction was guided by tactile feedback obtained during fibre pullback. Sticking of the radial fibre to the vein wall has the danger of causing excessive localized, non-circumferential thermal damage, which theoretically may lead to perforations or inadequate ablation. Hence, the rationale for reducing the power by 1 watt, which was not allowed more than twice. Interestingly sticking was predominantly found in Zones-2G and -3G for the GSV. In these zones power reduction was needed in 67.3% (Zone-2G; n = 99/147) and 38.7% (Zone-3G; n = 57/147) of procedures. This caused a deviation from the initial power settings, with the median power for Zone-2G being 3 W instead of expected 4 W (Table 1).

With this strategy, high early- (1 M; 98.9%) and mid-term (12 M; 93.7%) occlusion rates with few failures at 12 M (10/159;6.3%) were achieved. The occlusion rates are comparable to the current literature for EVLA with wavelength >1900 nm and radial emitting fibre, which shows 12-month occlusion rates of 88%, 95% and 98%, respectively [10,11,14]. A recent meta-analysis revealed an overall occlusion rate for EVLA, irrespective of wavelength and fibre type, to be 92% [25]. With this approach the incidence of postoperative complications at 1 month was low (LIP 2.7%, EHIT 1.6%). Viarengo et al. (1940 nm; radial fibre) reported higher rates of EHIT (4.8%) and pigmentation (9.7%) [11]. LIP (temporary and persistent) was lower in the current study as compared to the literature reporting the results for wavelength >1900 µm (6.25%, 7.3%, 3.77%, 2.5% and 6.2%) [10,11,12,13,14]. Persistent LIP at 12 M (0.7%) was also lower as compared to the literature. Lawson et al. and Jibiki et al. (1470 nm; radial fibre) reported persistent LIP in 2.9% and 1% of procedures respectively [6,7]. Setia et al. (1940 nm; radial fibre) reported persistent LIP in 2.2% of procedures [14]. Apart from LIP and MIP in one patient each, all the postoperative complications regressed at the 12-month follow-up. Regarding measurement and reporting of additional important end points, such as stump length, non-occlusion (NO) or recanalization (RC), no internationally accepted recommendations have been published in the literature [3]. Considering a stump length of >3 cm as EVLA failure in current study, a low mean stump length at 1 M and 12 M was achieved. Mendes-Pinto et al. (1940 nm; radial-fibre) regarded a stump length of >5 cm as pathological, and reported recanalization in 4/48 (8.3%) limbs within 4 weeks, which increased to 6/48 (12.5%) at 1 year [10]. Lawson et al. reported a stump length of 0.83 cm with RFO and 0.75 cm with EVLA (1470 nm; radial fibre) at 12 months [6]. Anatomically, the confluence of AVs and GSV usually lies within the first few centimetres from the SFJ [23], which makes it logical to think that a shorter stump length would yield durable EVLA results with low inguinal neoreflux [23].

Moreover, more than two decades of experience with endovenous ablation techniques has put a light on the importance of the AVs in initiating recurrent varicose veins [23,26]. In view of this, a preoperative duplex ultrasound should include and record the status (reflux, diameter) of the AVs in patients undergoing endovenous thermal ablation [23,27]. This was an additional improvement to our previous protocol [14]. The incompetent accessory veins similar to those in the literature tended to exhibit larger OD [26,28] and were treated with EVLA with radial fibre (Supplementary Table S3) [29,30].

The limitations of the current study lie in the single centre clinical application and lack of external validation. The authors are aware that the question of standardizing EVLA with reproducibility still remains open. Nonetheless the proposed four-zone guiding tool, accounting for the variable vein anatomy along its course, is a potential step towards standardizing dosimetry and documentation for EVLA. This strategy shows good mid-term results with minimal complications. A multi-center comparative study with long-term follow-up and comparison of the current four-zone guiding tool to older protocols is under planning. Such a guiding tool could improve the ability to analyse, compare and review different EVLA wavelengths and fibre types.

## Figures and Tables

**Table 1:** Dosimetry parameters for the great and small saphenous vein (GSV, SSV), guided by the four-zone guiding tool.

	GSV				SSV	
Parameters/Zone	Zone-1G	Zone-2G	Zone-3G	Zone-4G	Zone-1S	Zone-2S
Avg. Length (cm) range	8 4–11	22 9–33	16 5–27	10 2–20	8 4–11	18 10–30
Avg. LEED (J/cm) range	42 20–80	33 20–50	27 10–40	22 20–30	34 20–60	27 20–40
Median Power (W) range	4 2–5	3 2–5	3 1–4	2 1–3	3 1–4	2 1–3
Avg. Tumescence (mL) range	83 20–250	97 40–250	78 20–200	42 20–50	68 10–150	65 15–150
Avg. Tumescence per cm of vein (mL/cm)	10.4	4.4	4.9	4.2	8.5	3.6

LEED: linear endovenous energy density; GSV: great saphenous vein; SSV: small saphenous vein.

**Table 2:** Comparison of pre- and postoperative average diameters of the great saphenous vein (GSV) and small saphenous vein (SSV), with average percentage of reduction related to the pre-operative value and level of significance (*p*-value < 0.05 are significant). Outer diameter measurement G1–G7 and S1–S5 were in accordance with the protocol described elsewhere [21].

	Preoperative Average OD (mm) Range	FU 1 M Average OD (mm) Range	Average Reduction in OD 1 M (%)	FU 12 M Average OD (mm) Range	Average Reduction in OD 12 M (%)
G1	8.7 (3.9–18.3)	8.4 (3.2–15.2)	3.5% (*p* = 0.28)	7.6 (0–17.3)	12.6% (*p* = 0.08)
G2	7.3 (2.6–12.9)	6.4 (2.8–12.0)	12.3% (*p* = 0.33)	4.4 (0–8.9)	39.7% (***p* < 0.01**)
G3	5.8 (3.0–22.1)	4.7 (2.0–9.8)	18.9% (*p* = 0.06)	1.9 (0–5.8)	67.2% (***p* < 0.01**)
G4	5.8 (2.6–11.0)	4.5 (1.0–6.9)	22.4% (*p* = 0.07)	1.6 (0–8.5)	72.4% (***p* < 0.01**)
G5	5.0 (1.8–11.5)	3.9 (1.7–6.6)	22% (*p* = 0.06)	2.0 (0–6.5)	60% (***p* < 0.01**)
G6	4.3 (1.3–7.6)	3.4 (1.3–6.5)	20.9% (*p* = 0.06)	2.3 (0–7.0)	46.5% (***p* < 0.01**)
G7	3.0 (1.2–6.0)	2.4 (1.7–4.0)	20% (*p* = 0.06)	2.1 (0–4.3)	30% (***p* < 0.01**)
S1	6.1 (2.9–12.9)	6.1 (2.4–12.7)	0%	1.8 (0–8.0)	70% (***p* < 0.01**)
S2	5.6 (2.4–10.8)	4.8 (2.1–8.3)	14% (*p* = 0.12)	1.3 (0–6.1)	76.7% (***p* < 0.01**)
S3	5.2 (2.4–10.2)	4.3 (1.8–9.6)	17% (*p* = 0.84)	1.3 (0–5.6)	75% (***p* < 0.01**)
S4	4.8 (2.2–7.3)	4.1 (1.8–7.2)	14.5% (*p* = 0.61)	1.8 (0–5.6)	62.5% (***p* < 0.01**)
S5	3.0 (1.4–5.7)	3.0 (1.8–5.7)	-	2.1 (0–3.4)	30% (*p* = 0.9)

FU: follow-up. 1 M: 1 month. 12 M: 12 months.

**Table 3:** Summary of postoperative complications and duplex ultrasound findings at follow-up at 1 month (1 M) and 12 months (12 M), separated by the great saphenous vein (GSV) and small saphenous vein (SSV).

Parameters/Follow Up	Follow-Up 1 M		Follow-Up 12 M	
Median rVCSS Range	2 0–14		2 0–13	
	GSV (*n* = 147)	SSV (*n* = 38)	GSV (*n* = 126)	SSV (*n* = 33)
LIP	4*	0	1 *	0
MIP	4	0	1 **	0
EHIT	3	0	0	0
Hematoma thermal mechanical	9	1	0	0
	0	0	0	0
	9	1	0	0
DVT	0	0	0	0
Lymphocele	0	0	0	0
Skin Burns	0	0	0	0
Phlebitis	0	0	0	0
Wound infections	0	0	0	0
Non-Occlusion	2	0	NA	NA
Rekanalisation	NA	NA	6	2
Mean LSL (mm) range (mm)	9.7 0–200	3.7 0–20	12.3 0–100	4.7 0–52
Mean SSL (mm) range (mm)	6.4 0–200	3.0 0–14	8.9 0–100	4.1 0–51

* Laser-induced paresthesia was observed only below the knee. ** Was observed above and below the knees. N: number of EVLA procedures. EVLA: endovenous laser ablation. VCSS: revised venous clinical severity score. GSV: great saphenous vein. SSV: small saphenous vein. LIP: laser-induced paresthesia. MIP: mechanically induced paresthesia. EHIT: endovenous heat-induced thrombosis. DVT: deep vein thrombosis. LSL: longest stump length. SSL: shortest stump length. NA: not applicable.

## Data Availability

Data can be made available on request.

## Supplementary Materials

The following supporting information can be downloaded at: https://www.mdpi.com/article/10.3390/jcm12134313/s1, Figure S1: Postoperative Ultrasound demonstrating Endovenous heat induced thrombus (EHIT II), marked with red arrows, protruding in the deep vein. An imaginary line joining P1 and P2 represents the limit of the sapheno-femoral junction; Table S1: Patients’ characteristics and demographics; Table S2: Preoperative characteristics of the accessory veins; Table S3: Average incisions for phlebectomies combined with EVLA of GSV and SSV; Table S4: Management of cases developing EHIT EHIT. Refs [22,31,32] are cited in the Supplementary File.

## References

[1] Gloviczki P., Comerota A.J., Dalsing M.C., Eklof B.G., Gillespie D.L., Gloviczki M.L., Lohr J.M., McLafferty R.B., Meissner M.H., Murad M.H. (2011). The care of patients with varicose veins and associated chronic venous diseases: Clinical practice guidelines of the Society for Vascular Surgery and the American Venous Forum. J. Vasc. Surg..

[2] Pannier F., Noppeney T., Alm J., Breu F.X., Bruning G., Flessenkämper I., Gerlach H., Hartmann K., Kahle B., Kluess H. S2k—Leitlinie Diagnostik und Therapie der Varikose. AWMF Online 03/2019. https://register.awmf.org/assets/guidelines/037-018l_S2k_Varikose_Diagnostik-Therapie_2019-07.

[3] De Maeseneer M.G., Kakkos S.K., Aherne T., Baekgaard N., Black S., Blomgren L., Giannoukas A., Gohel M., de Graaf R., Hamel-Desnos C. (2022). Editor’s Choice—European Society for Vascular Surgery (ESVS) 2022 Clinical Practice Guidelines on the Management of Chronic Venous Disease of the Lower Limbs. Eur. J. Vasc. Endovasc. Surg..

[4] Vuylsteke M., De Bo T.H., Dompe G., Di Crisci D., Abbad C., Mordon S. (2011). Endovenous laser treatment: Is there a clinical difference between using a 1500 nm and a 980 nm diode laser? A multicenter randomised clinical trial. Int. Angiol..

[5] Jibiki M., Miyata T., Futatsugi S., Iso M., Sakanushi Y. (2016). Effect of the wide-spread use of endovenous laser ablation on the treatment of varicose veins in Japan: A large-scale, single institute study). Laser Ther..

[6] Lawson J.A., Gauw S.A., van Vlijmen C.J., Pronk P., Gaastra M.T., Tangelder M.J., Mooij M.C. (2018). Prospective comparative cohort study evaluating incompetent great saphenous vein closure using radiofrequency-powered segmental ablation or 1470-nm endovenous laser ablation with radial-tip fibers (Varico 2 study). J. Vasc. Surg. Venous Lymphat. Disord..

[7] Pavei P., Spreafico G., Bernardi E., Giraldi E., Ferrini M. (2021). Favorable long-term results of endovenous laser ablation of great and small saphenous vein incompetence with a 1470-nm laser and radial fiber. J. Vasc. Surg. Venous Lymphat. Disord..

[8] Schmedt C., Esipova A., Dikic S., Demhasaj S., Comsa F., Sroka R. (2014). Erste klinische Ergebnisse der Endovenösen Lasertherapie (ELT) mit Thulium (Tm) Laser (1940nm) und radialer Lichtapplikation. Vasomed.

[9] Schmedt C.-G., Esipova A., Dikic S., Setia A., Demhasaj S., Dieckmann T., Tipi M.-M., Sroka R. (2016). Endovenous Laser Therapy (ELT) Of Saphenous Vein Reflux Using Thulium Laser (Tm, 1940 nm) with Radial Fiber—One Year Results. Eur. J. Vasc. Endovasc. Surg..

[10] Mendes-Pinto D., Bastianetto P., Lyra L.C.B., Kikuchi R., Kabnick L. (2016). Endovenous laser ablation of the great saphenous vein comparing 1920-nm and 1470-nm diode laser. Int. Angiol..

[11] Viarengo L.M.A., Viarengo G., Martins A.M., Mancini M.W., Lopes L.A. (2017). Resultados de médio e longo prazo do tratamento endovenoso de varizes com laser de diodo em 1940 nm: Análise crítica e considerações técnicas. J. Vasc. Bras..

[12] Park I. (2019). Initial outcomes of endovenous laser ablation with 1940 nm diode laser in the treatment of incompetent saphenous veins. Vascular.

[13] Park I., Park S.-C. (2020). Comparison of Short-Term Outcomes Between Endovenous 1,940-nm Laser Ablation and Radiofrequency Ablation for Incompetent Saphenous Veins. Front. Surg..

[14] Setia A., Schmedt C.G., Beisswenger A., Dikic S., Demhasaj S., Setia O., Schmitz-Rixen T., Sroka R. (2021). Safety and efficacy of endovenous laser ablation (EVLA) using 1940 nm and radial emitting fiber: 3-year results of a prospective, non-randomized study and comparison with 1470 nm. Lasers Surg. Med..

[15] Sroka R., Weick K., Sadeghi-Azandaryani M., Steckmeier B., Schmedt C.-G. (2010). Endovenous laser therapy—Application studies and latest investigations. J. Biophotonics.

[16] Sroka R., Pongratz T., Siegrist K., Burgmeier C., Barth H.D., Schmedt C.G. (2013). Endovenous Laser Application Strategies to improve endoluminal energy application. Phlebology.

[17] Minaev V.P., Minaev N.V., Bogachev V.Y., Kaperiz K.A., Yusupov V.I. (2021). Endovenous laser coagulation: Asymmetrical heat transfer (modeling in water). Lasers Med. Sci..

[18] Minaev V.P., Minaev N.V., Bogachev V.Y., Kaperiz K.A., Yusupov V.I. (2022). Endovenous laser coagulation: Asymmetrical heat transfer and coagulation (modeling in blood plasma). Lasers Med. Sci..

[19] Sroka R., Weick K., Steckmaier S., Steckmaier B., Blagova R., Sroka I., Sadeghi-Azandaryani M., Maier J., Schmedt C.G. (2012). The ox-foot-model for investigating endoluminal thermal treatment modalities of varicosis vein diseases. ALTEX-Altern. Anim. Exp..

[20] Artemov S.A., Belyaev A.N., Bushukina O.S., Khrushchalina S.A., Kostin S.V., Lyapin A.A., Ryabochkina P.A., Taratynova A.D. (2021). Morphological changes of veins and perivenous tissues during endovenous laser coagulation using 2-μm laser radiation and various types of optical fibers. J. Vasc. Surg. Venous Lymphat. Disord..

[21] Setia A., Schmedt C.-G., Sroka R. (2022). Endovenous laser ablation using laser systems emitting at wavelengths > 1900 nm: A systematic review. Lasers Med. Sci..

[22] Vasquez M.A., Rabe E., McLafferty R.B., Shortell C.K., Marston W.A., Gillespie D., Meissner M.H., Rutherford R.B. (2010). Revision of the venous clinical severity score: Venous outcomes consensus statement: Special communication of the American Venous Forum Ad Hoc Outcomes Working Group. J. Vasc. Surg..

[23] Baccellieri D., Ardita V., Carta N., Melissano G., Chiesa R. (2020). Anterior accessory saphenous vein confluence anatomy at the sapheno-femoral junction as risk factor for varicose veins recurrence after great saphenous vein radiofrequency thermal ablation. Int. Angiol..

[24] Kabnick L.S., Sadek M., Bjarnason H., Coleman D.M., Dillavou E.D., Hingorani A.P., Lal B.K., Lawrence P.F., Malgor R.D., Puggioni A. (2021). Classification and treatment of endothermal heat-induced thrombosis: Recommendations from the American Venous Forum and the Society for Vascular Surgery. J. Vasc. Surg. Venous Lymphat. Disord..

[25] Malskat W.S.J., Engels L.K., Hollestein L.M., Nijsten T., van den Bos R.R. (2019). Commonly Used Endovenous Laser Ablation (EVLA) Parameters Do Not Influence Efficacy: Results of a Systematic Review and Meta-Analysis. Eur. J. Vasc. Endovasc. Surg..

[26] Proebstle T.M., Möhler T. (2015). A longitudinal single-center cohort study on the prevalence and risk of accessory saphenous vein reflux after radiofrequency segmental thermal ablation of great saphenous veins. J. Vasc. Surg. Venous Lymphat. Disord..

[27] Bush R.G., Bush P., Flanagan J., Fritz R., Gueldner T., Koziarski J., McMullen K., Zumbro G. (2014). Factors Associated with Recurrence of Varicose Veins after Thermal Ablation: Results of The Recurrent Veins after Thermal Ablation Study. Sci. World J..

[28] Joh J.H., Park H.-C. (2013). The cutoff value of saphenous vein diameter to predict reflux. J. Korean Surg. Soc..

[29] Myers K.A., Clough A., Tilli H. (2013). Endovenous laser ablation for major varicose tributaries. Phlebology.

[30] Müller L., Alm J. (2021). Feasibility and potential significance of prophylactic ablation of the major ascending tributaries in endovenous laser ablation (EVLA) of the great saphenous vein: A case series. PLoS ONE.

[31] Eklöf B., Rutherford R.B., Bergan J.J., Carpentier P.H., Gloviczki P., Kistner R.L., Meissner M.H., Moneta G.L., Myers K., Padberg F.T. (2004). Revision of the CEAP classification for chronic venous disorders: Consensus statement. J. Vasc. Surg..

[32] Augustin M., Debus E.S., Bruning G., Faubel R., Lohrberg D., Goepel L., Herberger K., Blome C. (2015). Development and Validation of a Short Version of the Freiburg Life Quality Assessment for Chronic Venous Disease (FLQA-VS-10). Wound Med..

