# Supplementary material for: Prospects of Endovenous Laser Ablation (EVLA) Standardization—Mid-Term Results of a Four-Zone Dosimetry Guiding Tool for 1940 nm Laser
Source: J Clin Med. 2023 Jun 27;12(13):4313. doi: 10.3390/jcm12134313 (PMC10342372; doi:10.3390/jcm12134313)
Supplement: Supplementary file 1 [file jcm-12-04313-s001.zip › jcm-2375035-supplementary figure.pdf]

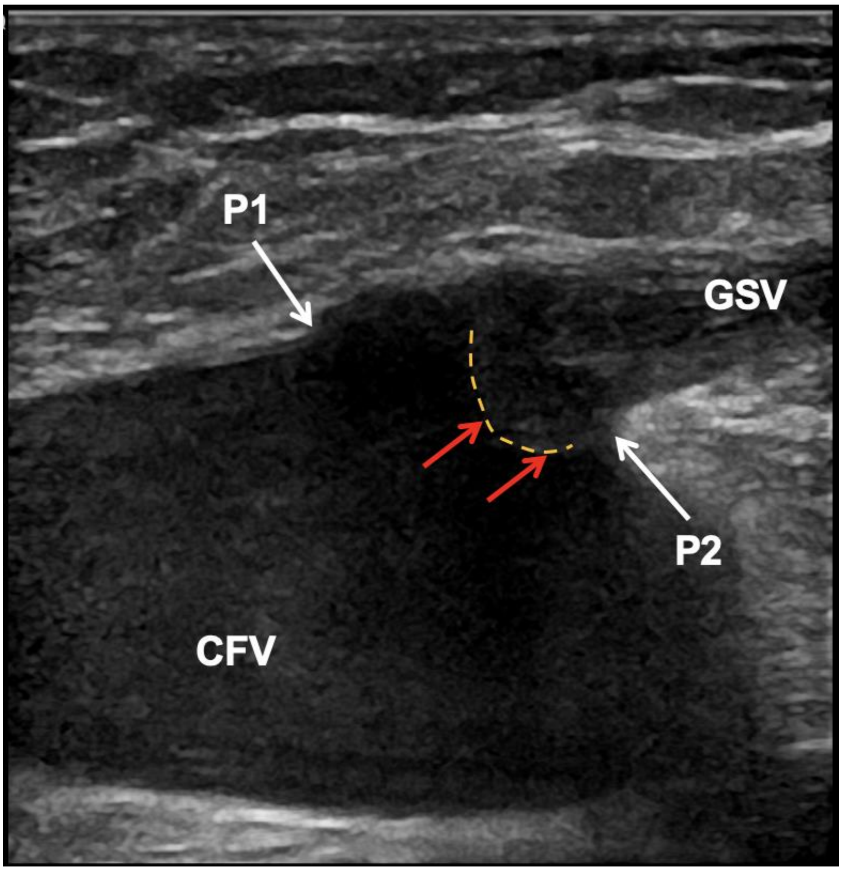

Supplemental Figure S1: Postoperative Ultrasound demonstrating Endovenous heat induced thrombus (EHIT II), marked with red arrows, protruding in the deep vein. An imaginary line joining P1 and P2 represents the limit of the sapheno-femoral junction.

P1: Proximal ostial point at the junction to the deep vein

P2: Distal ostial point at the junction to the deep vein.

GSV: Great saphenous vein
