# Supplementary material for: Prospects of Endovenous Laser Ablation (EVLA) Standardization—Mid-Term Results of a Four-Zone Dosimetry Guiding Tool for 1940 nm Laser
Source: J Clin Med. 2023 Jun 27;12(13):4313. doi: 10.3390/jcm12134313 (PMC10342372; doi:10.3390/jcm12134313)
Supplement: Supplementary file 1 [file jcm-12-04313-s001.zip › jcm-2375035-supplementary file.pdf]

Endovenous Laser Ablation
# - Procedure Documentation Protocol -

Copyright © Prof. Dr. med. Claus-Georg Schmedt, Schwäbisch Hall, Germany

Date of operation:

Surgeon: **Assistant:**

Patient details

Surname: First Name:

Date of Birth:

Sex: M / F / D

Use separate Protocol for each vein treated

Hight [cm]:________ ☐ General Anesthesia ☐ GSV ☐ right CEAP C: _______

Weight [Kg]:______ ☐ Spinal Anesthesia ☐ SSV ☐ left CEAP A (1-5) : __

ASA:_____________ ☐ LA +/- Sedation ☐ ___________(Others) rVCSS:_________

Laser wavelength/Fiber type:_________________________ FLQA-VS-10____

☐ Tumescence + LA ☐ Tumescence only ☐ No Tumescence Temp. Tume.:_______

Pre-OP OD	[mm]
G1/S1	
G2/S2	
G3/S3	
G4/S4	
G5/S5	
G6/S6	
G7/S7	

Pre-OP OD	Identified [Y/N]	OD3 [mm]	Reflux [Y/N]
AASV			
PASV			

ZONE	Length [cm]	Tume. [ml]	P [Watt]	V [mm/s]	LEED [J/cm]
Zone 1G					
Zone 2G					
Zone 3G/1S					
Zone 4G/2S					

Adjunct Procedures ipsilateral: ☐ NONE

☐ Miniphlebectomies Thigh, Incisions [n]:___

☐ Miniphlebectomies Leg, Incisions [n]:______

☐ Crossectomy GSV ☐ Stripping GSV

☐ Crossectomy SSV ☐ Stripping SSV

☐ Perforator Lig. Thigh,Incisions [n]:__

☐ Perforator Lig. Leg, Incisions [n]:___

☐ EVLA GSV

☐ EVLA SSV

Adjunct Procedures contralateral ☐ NONE

☐ Miniphlebectomies Thigh, Incisions [n]:___

☐ Miniphlebectomies Leg, Incisions [n]:______

☐ Crossectomy GSV ☐ Stripping GSV

☐ Crossectomy SSV ☐ Stripping SSV

☐ Perforator Lig. Thigh,Incisions [n]:__

☐ Perforator Lig. Leg, Incisions [n]:___

☐ EVLA GSV

☐ EVLA SSV

Copyright © Prof. Dr. med. Claus-Georg Schmedt, Schwäbisch Hall, Germany
Copying and reproduction in any form for commercial purposes not permitted

Endovenous Laser Ablation
# - Follow-up Protocol -

Copyright © Prof. Dr. med. Claus-Georg Schmedt
Schwäbisch Hall, Germany

☐ W1 ☐ M1 ☐ M6 ☐ Y1 ☐ Y2 ☐ Y_x

Date of Follow-Up:

Examiner:

Patient details

Surname: First Name:

Date of Birth:

Sex: M / F / D

Use separate Protocol for each vein treated

Date of Operation: ☐ GSV ☐ SSV ☐ right ☐ left

Laser Wavelength:_______________

Fiber Type:_______________________

Max. intraoperativ pain intensity [NAS 0-10]: ____

p.o. Analgesia i.v. (nr. of doses) [n]: _______ Inability to work (Days p.o.) [n]: _____

p.o. Analgesia oral (nr. of doses) [n]: ______ Return to daily activities (Days p.o.) [n]: ____

Clincal status: CEAP: C_____ rVCSS:_______ FLQA-VS-10____

Pain intensity, Thigh [NAS 0-10]: _____ Pain intensity, Leg [NAS 0-10]:______

☐ Paresthesia LIP, Thigh [cm^2]:______ ☐ Paresthesia MIP, Thigh [cm^2]:_______

☐ Paresthesia LIP, Leg [cm^2]:_________ ☐ Paresthesia MIP, Leg [cm^2]:_________

☐ Hematoma EVLA, Thigh [cm^3]:_____ ☐ Hematoma Phlebectomy, Thigh [cm^3]:___

☐ Hematoma EVLA, Leg [cm^3]:______ ☐ Hematoma Phlebectomy, Leg [cm^3]:_____

Duplex:

Longest stump length (LSL) [mm]: ___ Reflux: ☐ no ☐ yes
[Maximum length of the non occluded lumen of the treated vein]

Shortest stump length(SSL) [mm]: __________
[Shortest distance between the SFJ/SPJ and the occluded vein]

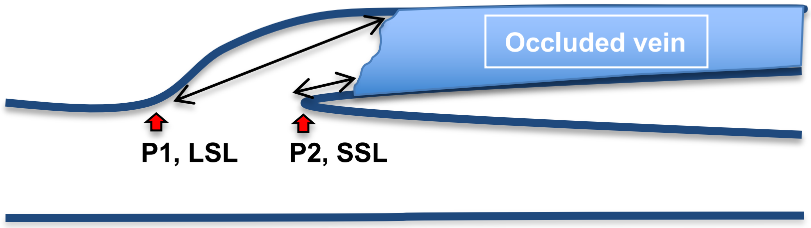

AASV patent: ☐ no ☐ yes Reflux: ☐ no ☐ yes

PASV patent: ☐ no ☐ yes Reflux: ☐ no ☐ yes

Other patent veins: ☐ no ☐ yes Reflux: ☐ no ☐ yes

OD (mm): AASV:____ PASV:____ Other patent veins:____

Recanalisation peripheral [cm]: ____ Reflux: ☐ no ☐ yes

Post-OP OD	[mm]
G1/S1	
G2/S2	
G3/S3	
G4/S4	
G5/S5	
G6/S6	
G7/S7	

Further comments:

Copyright © Prof. Dr. med. Claus-Georg Schmedt, Schwäbisch Hall, Germany
Copying and reproduction in any form for commercial purposes not permitted

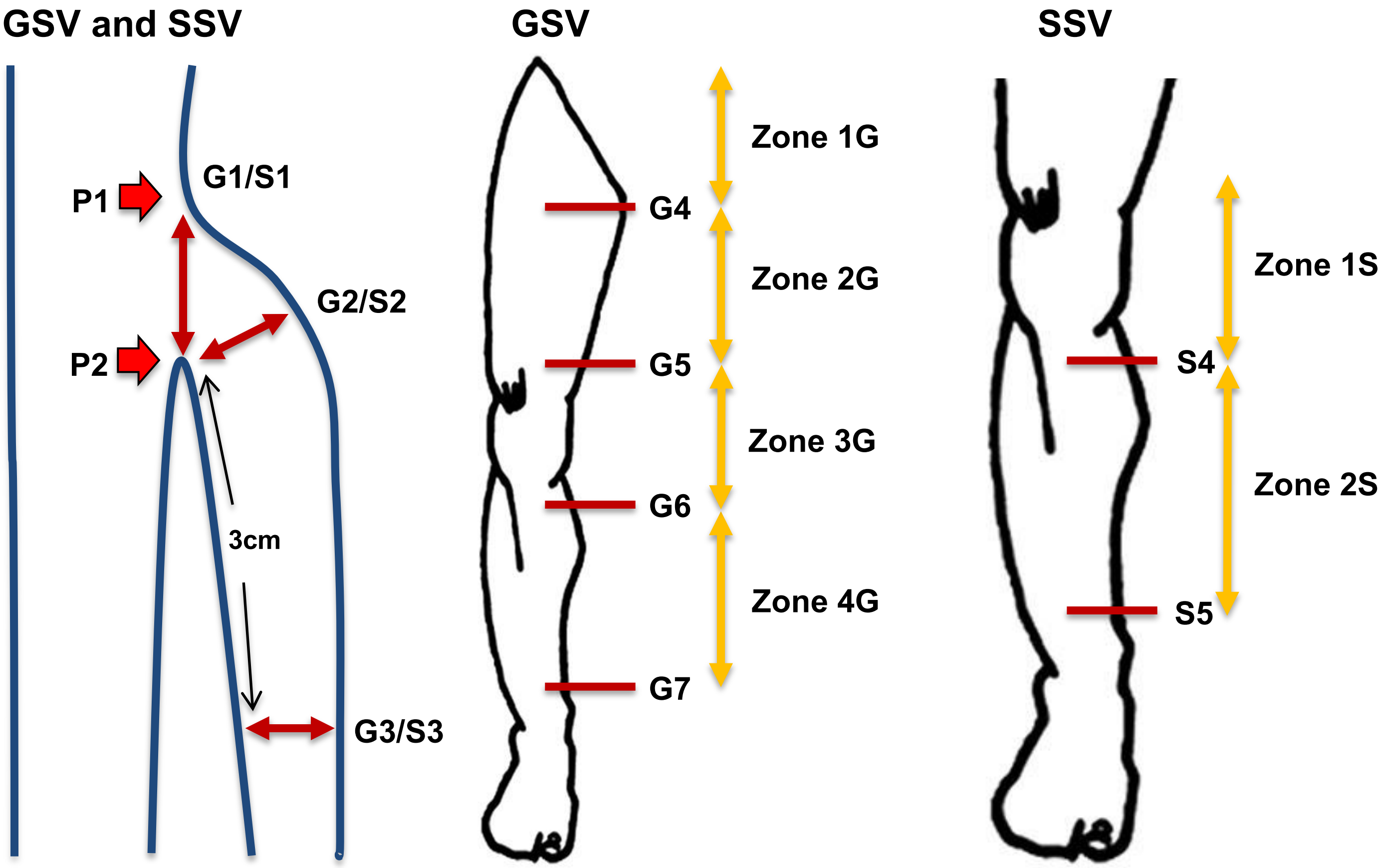

4-Zone Model

Zone-1G extended from inguinal ligament to the apex of femoral triangle (level of perineum)
Zone-2G from the apex of femoral triangle to the upper border of patella
Zone-3G from patella to tibial tuberosity
Zone-4G extended from the tibial tuberosity to ankle joint for GSV
Zone-1S extended from SPJ to tibial tuberosity for the SSV
Zone-2S extended from the tibial tuberosity to ankle joint for SSV

Legend:

GSV: Great saphenous vein, SSV: Small saphenous vein, AASV: Anterior accessory saphenous vein, PASV: Posterior accessory saphenous vein, CEAP: CEAP classification, rVCSS: Revised venous clinical severity score, FLQA-VS-10: Freiburg life quality assessment for chronic venous diseases, LA: Local anesthesia, Pre-OP: Pre-operative, OD: Outer diameter, Tume.: Tumescence, P: Power, V: Pullback velocity, LEED: Linear endovenous energy density, Lig.: Ligature.

W1: Follow-up within 1 week postoperative, M1: at 1 month, M6: at 6 months, Y1: at 1 year, Y2: at 2 years, p.o.: Postoperative, i.v.: Intravenous, NAS: Numerical analogue scale (0-10), LIP: Laser induced paresthesia, MIP: Mechanical induced paresthesia, AASV: Anterior accessory saphenous vein, PASV: Posterior accessory saphenous vein.

G1: Outer diameter of GSV at the junction with the deep vein, G2: Outer diameter of GSV measured from distal ostial point (P2) at sapheno-femoral junction (SFJ), perpendicular to the central vessel axis, G3: Outer diameter of GSV 3 cm peripheral to the junction to the deep vein, G4: Outer vein-diameter at the level of perineum for GSV, G5: Outer vein-diameter at the upper border of Patella for GSV, G6: Outer vein-diameter at the level of tibial tuberosity for GSV, G7: Outer vein-diameter proximal to the medial malleolus for GSV, S1: Outer diameter of SSV at the junction with the deep vein, S2: Outer diameter of SSV measured from distal ostial point (P2) at sapheno-poplitea junction (SPJ), perpendicular to the central vessel axis S3: Outer diameter of SSV 3cm peripheral to the junction to the deep vein, S4: Outer vein-diameter at the level of tibial tuberosity for SSV, S5: Outer vein-diameter proximal to the lateral malleolus for SSV. P1: Proximal ostial point at the sapheno-femoral (SFJ) or sapheno-poplitea junction (SPJ), P2: Distal ostial point at the SFJ/SPJ.

Literature:

Eklöf B, Rutherford RB, Bergan JJ et al. Revision of the CEAP classification for chronic venous disorders: consensus statement. J Vasc Surg. 2004; 40(6):1248–1252. doi: 10.1016/j.jvs.2004.09.027.
Vasquez MA, Rabe E, McLafferty RB et al. American Venous Forum Ad Hoc Outcomes Working Group. Revision of the venous clinical severity score: venous outcomes consensus statement: special communication of the American Venous Forum Ad Hoc Outcomes Working Group. J Vasc Surg. 2010 Nov; 52(5):1387-96. doi: 10.1016/j.jvs.2010.06.161. Epub 2010 Sep 27. PMID: 20875713.
Augustin M, Debus ES, Bruning G et al. Development and Validation of a Short Version of the Freiburg Life Quality Assessment for Chronic Venous Disease (FLQA-VS-10). Wound Medicine. 2015;8:31-35.

Copyright © Prof. Dr. med. Claus-Georg Schmedt, Schwäbisch Hall, Germany
Copying and reproduction in any form for commercial purposes not permitted
