# Supplementary material for: Prospects of Endovenous Laser Ablation (EVLA) Standardization—Mid-Term Results of a Four-Zone Dosimetry Guiding Tool for 1940 nm Laser
Source: J Clin Med. 2023 Jun 27;12(13):4313. doi: 10.3390/jcm12134313 (PMC10342372; doi:10.3390/jcm12134313)
Supplement: Supplementary file 1 [file jcm-12-04313-s001.zip › jcm-2375035-supplementary tables.pdf]

		Females (n=92)	Males (n=60)
Mean age (years)<br>range		60<br>22-85	58<br>24-82
Mean Body Mass Index (kg/m^2)<br>range		26.3<br>18.4 – 39.1	28.7<br>17.9- 48.1
Distribution according to the CEAP Classification (percentage)			
CEAP	C1	1 (0.5%)	
	C2	35 (19%)	
	C3	100 (54%)	
	C4	36 (20%)	
	C5	5 (2.2%)	
	C6	8 (4.3%)	
	Total	185 (100%)	

Supplementary Table S1: Patients' characteristics and demographics.

	AASV	Avg. OD (mm)<br>range		PASV	Avg. OD (mm)<br>range
Incompetent	22<br>(18.8%)	4.2<br>2.2-6.3		3<br>(2.7%)	4.6<br>3.5-6.5
Competent	66<br>(56.4%)	2.7<br>1.2-5.0		63<br>(53.8%)	3.2<br>1.2-5.3
Not identifiable	29<br>(24.8%)	-		51<br>(43.5%)	-

Total	117 (100%)	3.1 1.2-6.3		117 (100%)	3.4 1.2-6.5

Supplementary Table S2: Preoperative characteristics of the accessory veins

OD: Outer diameter

AASV: Anterior accessory saphenous veins

PASV: Posterior accessory saphenous veins

Avg.: Average

(+): Present

(-): Absent

	Average incisions above knee (range)	Average incisions below knee (range)
Accompanying GSV EVLA	5 (0-20)	7 (0-13)
Accompanying SSV EVLA	3 (0-8)	6 (0-12)
Total	5(0-20)	7 (0-13)

Supplementary Table S3: Average incisions for phlebectomies combined with EVLA of GSV and SSV.

EVLA: Endovenous laser ablation

GSV: Great saphenous vein

SSV: Small saphenous vein

	EHIT Category	EHIT-Therapy	Resolution within (weeks post procedure)
Case 1	I	Rivaroxaban 15mg OD for 3 Weeks	3
Case 2	II	Enoxaparin 1mg/kg BD for 1Week	1
Case 3	II	Enoxaparin 1mg/kg BD for 1Week	1

Supplementary Table S4: Management of cases developing EHIT

EHIT: Endovenous heat induced thrombosis.

OD: Once in a day

BD: Twice daily
